# Replication and transcription of human papillomavirus type 58 genome in *Saccharomyces cerevisiae*

**DOI:** 10.1186/1743-422X-7-368

**Published:** 2010-12-15

**Authors:** Jing Li, Xiao Wang, Juan Liu, Hong Wang, Xiao-Li Zhang, Wei Tang, Yun-Dong Sun, Xin Wang, Xiu-Ping Yu, Wei-Ming Zhao

**Affiliations:** 1 Department of Medical Microbiology, Shandong University School of Medicine, Jinan, Shandong, 250012, PR China; 2 Department of Pathology, Shandong University School of Medicine, Jinan, Shandong, 250012, PR China

## Abstract

**Background:**

To establish a convenient system for the study of human papillomavirus (HPV), we inserted a *Saccharomyces cerevisiae *selectable marker, Ura, into HPV58 genome and transformed it into yeast.

**Results:**

HPV58 genome could replicate extrachromosomally in yeast, with transcription of its early and late genes. However, with mutation of the viral E2 gene, HPV58 genome lost its mitotic stability, and the transcription levels of E6 and E7 genes were upregulated.

**Conclusions:**

E2 protein could participate in viral genome maintenance, replication and transcription regulation. This yeast model could be used for the study of certain aspects of HPV life cycle.

## Background

Human papillomaviruses are small circular DNA viruses that infect epithelial cells and normally replicate as nuclear plasmids. The life cycle of papillomavirus is tightly linked to epithelial differentiation [1]. Among the high-risk HPV types associated with cervical cancer, human papillomavirus type 58 (HPV58) plays a more prominent role in Asian countries. HPV58 has been found in 5.9% of cervical cancer patients in China [2], with an unusually high prevalence in cervical cancer patients in specific areas of China: 33.3% in Hong Kong [3] and 16.3% in Shanghai [4]. Despite the availability for biological study of few cell lines containing the DNA of high-risk HPVs such as 16, 18 and 31, no cell lines or animal models containing HPV58 have been established.

*Saccharomyces cerevisiae *is a species of budding yeast. The cellular mechanism required for DNA replication in S. cerevisiae is similar to that in human cells [5]. Studies have shown yeast to be a versatile organism for the study of viruses. Many types of DNA and RNA viruses, including HPVs, can directly replicate in yeast [6]. Although HPV6, 16 and 31 can replicate stably in yeast cells as nuclear plasmids [7,8], whether HPV58 genome can replicate stably in yeast and whether the viral genes can be transcribed in yeast are unknown.

In the present study, we first explored the replication and transcription of HPV58 genome in the yeast system and investigated the function of E2 on vrial DNA replication and transcription. We found that HPV58 genome could replicate stably as an episome, with transcription of its early and late genes in yeast. However, with mutation of the E2 gene, HPV58 genome lost its mitotic stability and the transcription levels of E6 and E7 genes were upregulated. Thus, E2 protein can facilitate the replication and maintenance of HPV58 DNA and regulate viral gene transcription in yeast cells.

## Methods

### DNA construction

#### HPV58-Ura

The Ura gene was amplified from *S. cerevisiae *plasmid pRS316 with the sense (5'-GATCCACCGGTGGCAGATTGTACTGAGAGTG-3') and anti-sense (5'-CTAGCACCG GTGTAGTATACATGCATTTAC-3') primers containing the *Sgr*A I site (underlined). The PCR-produced Ura was subcloned into the L2 open reading frame (ORF) of pEGFPN1-HPV58 to construct a pEGFPN1-HPV58-Ura plasmid. The HPV58-Ura was released from pEGFPN1-HPV58-Ura by *Bgl *II digestion and recircularized by T4 DNA ligase for yeast transformation.

#### HPV58-Ura-E2 mutant (HPV58-Ura-E2mt)

A 1236 bp cassette was PCR amplified from pEGFPN1-HPV58 with the sense (5'-C*CACCAGGTG***TAATGATGA**TTGGTAGCATC AAAGAC-3') and anti-sense (5'-CATAC*CACCATGTG*CAGAACCA-3') primers containing the *Dra *III site (underlined) and three stop codons (bold). The cassette was then substituted for the *Dra *III digested fragment (2924-4145 bp in HPV58 genome) in the pEGFPN1-HPV58-Ura plasmid to construct a pEGFPN1-HPV58-Ura-E2mt plasmid with three stop codons (2927-2932 bp) in the E2 ORF (2753-3829 bp). The HPV58-Ura-E2mt was released by *Bgl *II and recircularized for transformation.

#### pDBLeu-E2

The E2 protein expression plasmid was constructed as follows: E2 ORF was PCR amplified with the sense primer (5'-CCAAGCTTGAAAATTGGAAATCCT-3') containing the *Hind *III site (underlined) and anti-sense primer (5'-CTGCTAGCTTA**CAAGT CTTCTTCAGAGATCAACTTCTGTTC**CAATGACATAACACCAGTACT-3') containing the *Nhe *I site (underlined) and a cMyc tag (bold). The E2 PCR product was subcloned into pDBLeu to construct the pDBLeu-E2.

### Yeast transformation

*S. cerevisiae *strain W303-1B (*MAT*α *leu2-3 leu2-112 trp1-1 ura3-1 his3-11 his3-15 ade2-1 can1-100*) were transformed with different sets of plasmids: 1). pRS316; 2). HPV58-Ura; 3). HPV58-Ura-E2mt; 4). HPV58-Ura-E2mt and pDBLeu (HPV58-Ura-E2mt/pDBLeu); 5). HPV58-Ura-E2mt and pDBLeu-E2 (HPV58-Ura-E2mt/pDBLeu-E2). The transformed yeast were spotted on selective medium plates for 3-5 days. Single colonies were selected and cultured in yeast extract/peptone/dextrose (YPD) or synthetic complete (SC) dropout media (Clontech) for further analysis.

### Quantitative PCR (qPCR)

Yeast were cultured in 10 ml selective medium to an OD600 of 1.0 and yeast DNA was isolated as described [7]. The DNA was analysed by absolute qPCR with primers specific for E1 (sense: 5'-CTGCAATGGATGACCCTGAAG-3'; anti-sense: 5'-CCACTATCGTCTGCTGTTTCGT-3', amplicon: 136 bp, 878-1013 bp). Yeast 18S rDNA was used as internal control (sense: 5'-TTGTGCTGGCGATGGTTCA-3'; anti-sense: 5'-TGCTGCCTTCCTTGGATGTG-3', amplicon: 152 bp). A standard curve was generated by amplification of a serial dilution of pEGFPN1-HPV58-Ura.

### Southern blot analysis

Yeast harboring HPV58-Ura and HPV58-Ura-E2mt/pDBLeu-E2 were grown in 25-ml selective medium overnight to yield an OD600 of 1.0. HPV58-Ura-E2mt transformed yeast were cultured in 25 ml selective medium for 3 days to yield an OD600 of 0.2, because of the poor growth in selective medium. Yeast DNA was isolated and digested with *Xho *I (no cut on HPV58 genome), *Bgl *II (1 cut), *Hpa *I (1 cut) or *Dpn *I (9 cuts) for 24 hr. *Dpn *I can digest methylated DNA isolated from bacteria only. The DNA was electrophoresed, blotted onto nylon membrane (Roche) and probed with an L1 specific mRNA probe labeled with digoxigenin by *in vitro *transcription according to the manufacturer's instructions (DIG RNA Labeling Kit, Roche).

### Western blot analysis

Yeast protein was prepared from yeast harboring pDBLeu-E2, pDBLeu, and untransformed yeast as previously described [9] Protein samples were separated by SDS-PAGE electrophoresis and transferred to nitrocellulose membrane. Then the membrane was blocked with 3% BSA in PBST and immunoblotted with antibody against cMyc (Santa Cruz). Membrane was washed and incubated with HRP conjugated secondary antibody. Chemilucent *ECL *Detection System (Millipore) was used to detect the signals according to the manufacturer's instruction.

### HPV58 genome stability assay

The DNA stability assay was performed as previously described [10]. Transformed yeast were first grown in selective medium to mid-log phase and diluted to an OD600 of 0.1 in new cultures containing non-selective medium. The cultures were grown for 17 hr (10 cell generations). The cultures at either 0 or 10 generations were diluted to an OD600 of 0.1. Aliquots of 5 μl were spotted to selective and non-selective media. After 3 days of growth, the percentage of colonies containing viral DNA was determined by the ratio of the number of colonies on selective medium to those on non-selective medium. The percentage of DNA loss per cell generation was calculated by subtracting the percentage DNA retained after 10 generations from that at 0 generation and divided by the total number of generations.

### RNA extraction, RT-PCR and quantitative RT-PCR (qRT-PCR)

Yeast were cultured in 10 ml selective medium to an OD600 of 1.0 or 0.2 (HPV58-Ura-E2mt transformed yeast). Yeast DNA and RNA were isolated from the same samples. Yeast total RNA was isolated as previously described [11] and digested by DNase I (Fermentas) to remove the contaminating DNA. PCR involved use of DNase I-treated RNA as a template to ensure the complete digestion of contaminating DNA in RNA samples.

The DNase I treated RNA was analysed by RT-PCR and absolute qRT-PCR with primers specific for E1 (sense: 5'-CTGCAATGGATGACCCTGAAG-3'; anti-sense: 5'-CCACTATCGTCTGCTGTTTCGT-3', amplicon: 136 bp, 878-1013 bp), E2 (sense: 5'-GACAAAGCGACGACGACT-3'; anti-sense: 5'-GTCGTTGTGTTTCCGTTGT-3', amplicon: 335 bp, 3427- 3761 bp), E6 (sense: 5'-ACTATGTTCCAGGACGCAGAG-3'; anti-sense: 5'-ACCTCAGATCGCTGCAAAG-3', amplicon: 128 bp, 107-234 bp), E7 (sense: 5'-GACGAGGATGAAATAGGCTTG-3'; anti-sense:5'-CGTCGGTTGTTGTACTGTTGA-3', amplicon: 133 bp, 670-802 bp), L1 (sense: 5'-CTTGAAATAGGTAGGGGACAG-3'; anti-sense: 5'-CAATGGAGGACAATCAGTAGC-3', amplicon: 249 bp, 5958-6206 bp) and L2 (sense: 5'-CATAGTGACATATCGCCTGCTC-3'; anti-sense: 5'-AGCCCCTATTTGCT TTCCAC-3', amplicon: 153 bp, 5051-5203 bp) respectively. Standard curves were generated by amplification of a serial dilution of pEGFPN1-HPV58-Ura.

Relative qRT-PCR was ued to compare the transcription levels of E6 and E7 genes with or without E2 protein. Because HPV58 genomes have different replication efficiency in HPV58-Ura-, HPV58-Ura-E2mt- and HPV58-Ura-E2mt/pDBLeuE2-transformed yeast, we first compared the relative replication levels of the HPV58 genome in different transformants by qPCR. The E1 primers described previously and 18S rDNA primers (sense:5'-TTGTGCTGGCGATGGTTCA-3'; anti-sense: 5'-TGCTGCCTTCCTTGGATGTG -3', amplicon: 152 bp, as internal control) were used in qPCR. Then qRT-PCR was performed to analyze the relative transcription levels of E6 and E7 genes of HPV58-Ura, HPV58-Ura-E2mt and HPV58-Ura-E2mt/pDBLeu-E2 in yeast, with the 18S rRNA as internal control. The value of relative transcription levels was divided by the value of DNA relative replication levels to standardize the transcription templates.

## Results

### Episomal replication of HPV58 in yeast

Viral DNA copy number was detected by qPCR from five continuous yeast passages as shown in Table 1. The viral DNA copy number in per microliter of yeast DNA is relatively consistent in the five continous passages. Averagely, there are 3-5 copies of viral DNA in per yeast cell.

**Table 1:** HPV58 genome copy number in cotinuous 5 passages:

Passage	viral DNA copies/μg yeast DNA*
P6	5.13×10^7 ^± 4.85×10^6^
P7	4.09×10^7 ^± 3.38×10^6^
P8	6.35×10^7 ^± 4.45×10^6^
P9	5.93×10^7 ^± 2.14×10^6^
P10	7.09×10^7 ^± 4.67×10^6^

DNA was isolated from yeast culture from passage 5 to passage 10. Viral DNA copies were quantitated by quantative PCR. The standard curve was generated by serially diluted pEGFPN1-HPV58-Ura. There is 3-5 copies of viral DNA in per yeast cells.

*Values are mean ± standard deviation.

**Figure 1:**
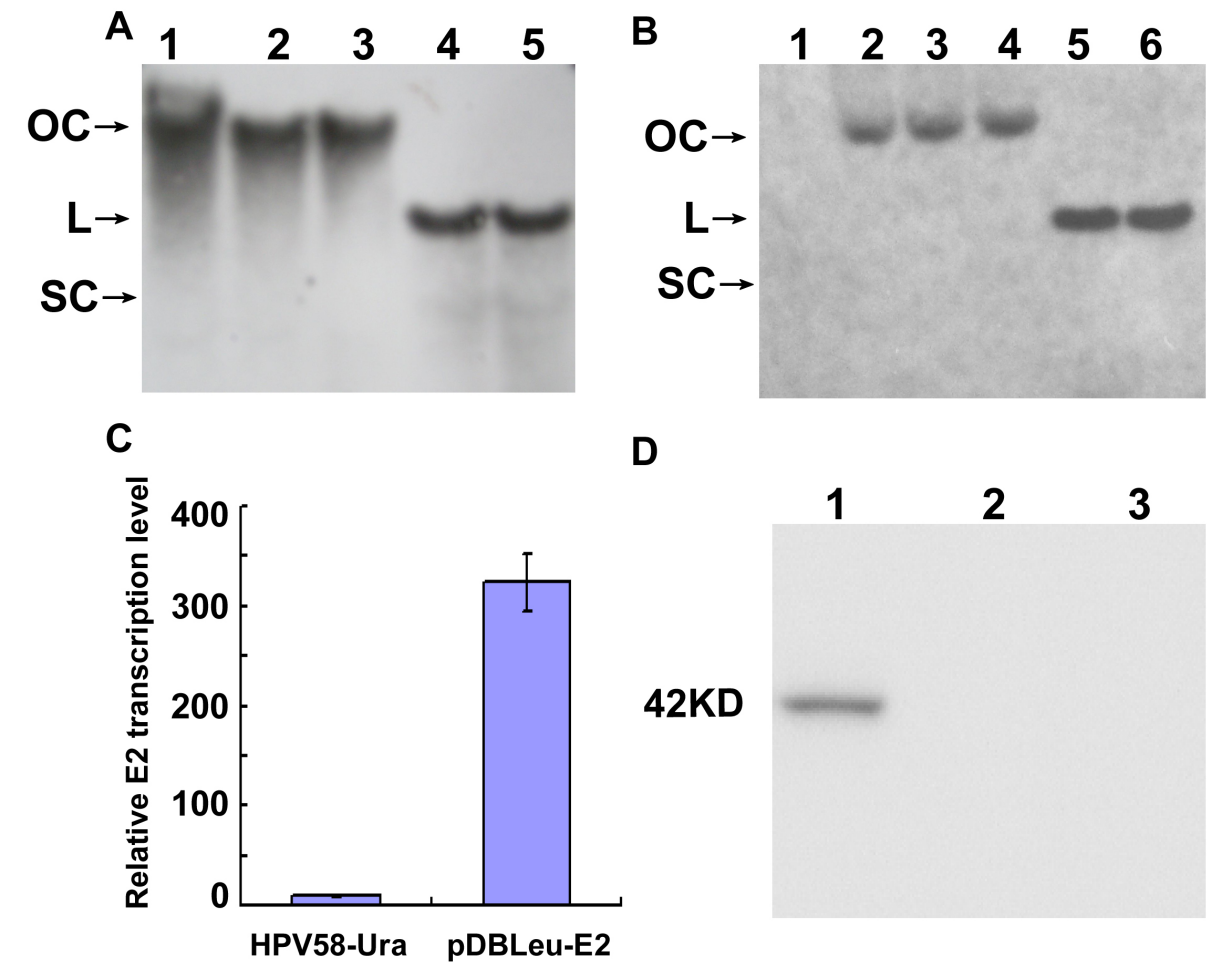
**Replication of HPV 58 genome in *S. cerevisiae***. **A **and** B**: Episomal replication of HPV58 genome in *S. cerevisiae*. Yeast DNA underwent Southern blot with an L1 specific mRNA probe. **A**, (DNA isolated from yeast harboring HPV58-Ura) Lane 1: yeast DNA without enzyme digestion; lane 2-5: yeast DNA digested with *Dpn *I, *Xho *I, *Bgl *II and *Hpa *I. **B**, Lane 1: DNA isolated from HPV58-Ura-E2mt transformed yeast and treated with *Dpn *I; lanes2-6 (yeast DNA isolated from HPV58-Ura-E2mt/pDBLeu-E2 transformed yeast), lane 2: DNA without enzyme digestion; lanes 3-6: yeast DNA digested with *Dpn *I, *Xho *I, *Bgl *II and *Hpa *I separately. Arrows show the positions of open-circle (OC), linear (L) and supercoiled (SC) forms of episomal HPV58-Ura or HPV58-Ura-E2mt. **C**. qRT-PCR to compare the E2 transcription levels in yeast cells harboring HPV58-Ura or pDBLeu-E2. **D**. Expression of E2 protein in yeast. Yeast protein was prepared from yeast harboring pDBLeu-E2 (lane 1), pDBLeu (lane 2), and untransformed yeast (lane 3).

Southern blot was performed to investigate the replication form of HPV58 genome in yeast. As shown in Figure 1A and 1B, when yeast DNA was treated with *Dpn *I or restriction enzymes which have no recognition site in HPV58 genome, the main form of HPV58 genome was open circular (OC). The vortex process during yeast DNA isolation may disrupt the supercoiled plasmid (SC) and lead to the formation of OC plasmid. Yeast DNA digested by a single-cut restriction enzyme revealed only a single band representing the linear form of HPV58 (Figure 1A). Therefore, HPV58 genome could replicate episomally in yeast.

Furthermore, no band was detected in the *Dpn *I treated DNA isolated from HPV58-Ura-E2mt transformed yeast (Figure 1B, lane 1), which indicates that the E2 gene mutation induced instability and decreased the replication level of viral DNA. Transformation of pDBLeu-E2 into yeast restored the mitotic stability of HPV58-Ura-E2mt and clear bands were detected (Figure 1B, lane 2-6). We have tried to detect and compare the expression levels of E2 protein from HPV58-Ura and pDBLeu-E2 with anti-HPV16 E2 antibody, but no bands were detected. The transcription levels of E2 from HPV58-Ura and pDBLeu-E2 were compared with qRT-PCR, the mRNA level of E2 from pDBLeu-E2 is 323 folds to that from HPV58-Ura (Figure 1C). Furthermore, we detected E2 protein in pDBLeu-E2 transformed yeast with anti-cMyc antibody as shown in Figure 1D. Therefore, E2 protein could facilitate viral genome replication and maintenance.

### Role of E2 protein in maintaining HPV58 genome in yeast

**Figure 2:**
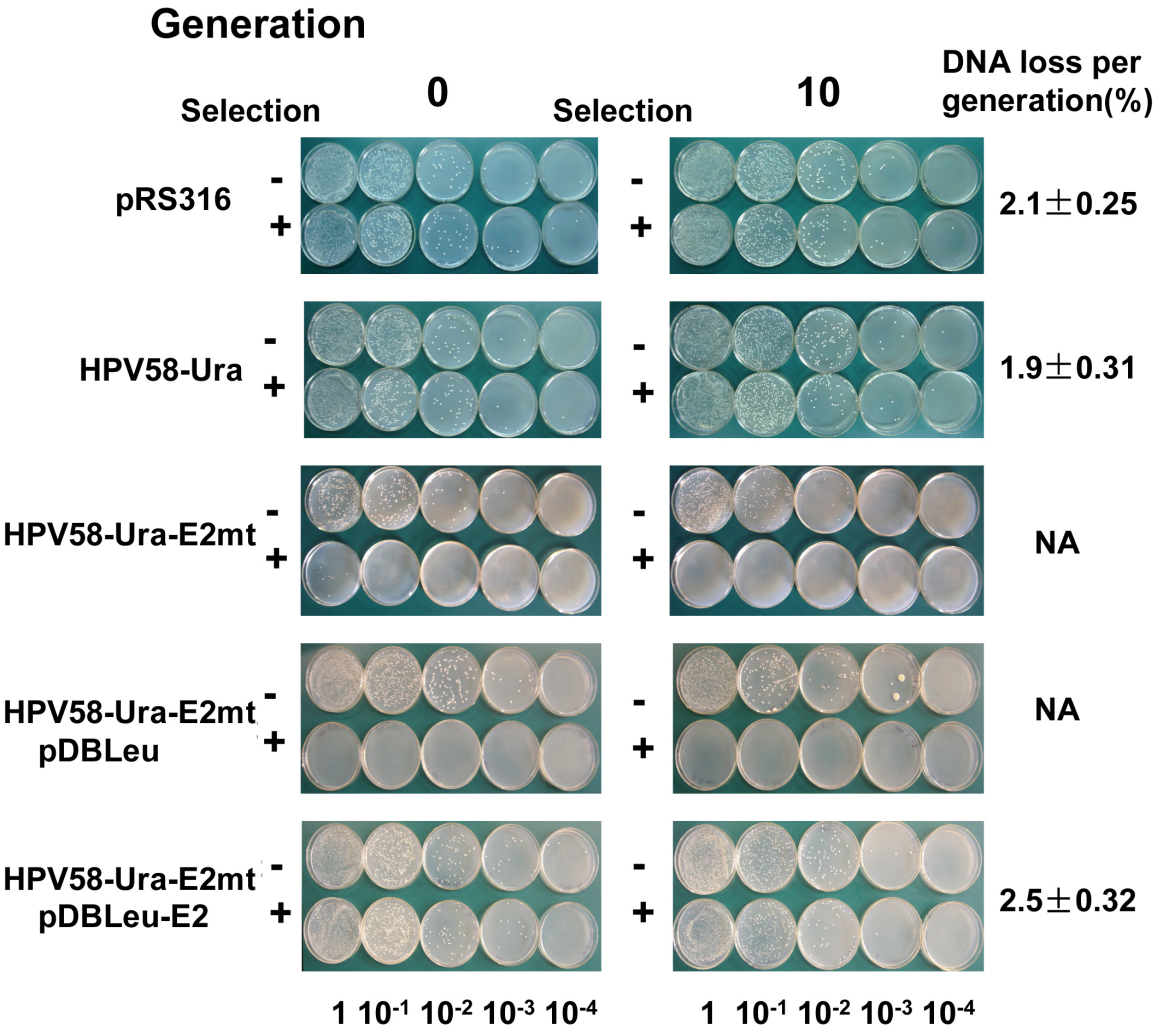
**DNA stability assay of HPV58 genome**. Yeast containing different recombinant HPV58 genomes were grown under non-selective conditions for 10 cell generations. After the incubation period, the cell cultures were serially diluted from 1 to 10^-5^. Equal volumes of each of the dilutions were spotted onto selective (+) and non-selective (-) media. The percentage of DNA loss per cell generation was calculated by subtracting the percentage DNA retained after 10 generations from that at 0 generation and divided by the total number of generations. Values on the right are the means of three independent experiments. NA: non-available.

DNA stability assay was performed to investigate the maintenance function of E2 protein on HPV58 genome. The DNA loss per cell generation for HPV58-Ura (1.9%) was comparable to that of the yeast plasmid pRS316 (2.1%) which contains centromeric elements (CEN) (Figure 2). Thus, the HPV58 genome was as stable as a yeast CEN containing plasmid.

Yeast harboring HPV58-Ura-E2mt grew poorly in selective medium, with no colony generated on the selective plates at G0 and G10. However, with transformation of pDBLeu-E2 and re-expression of E2 protein (Figure 1C, D), the HPV58 genome restored its mitotic stability to a DNA loss rate of 2.5% per cell generation (Figure 2). Therefore, E2 protein is critical to the mitotic stability of the HPV58 genome in yeast.

### Suppression of E6 and E7 transcription by E2 protein in yeast

**Figure 3:**
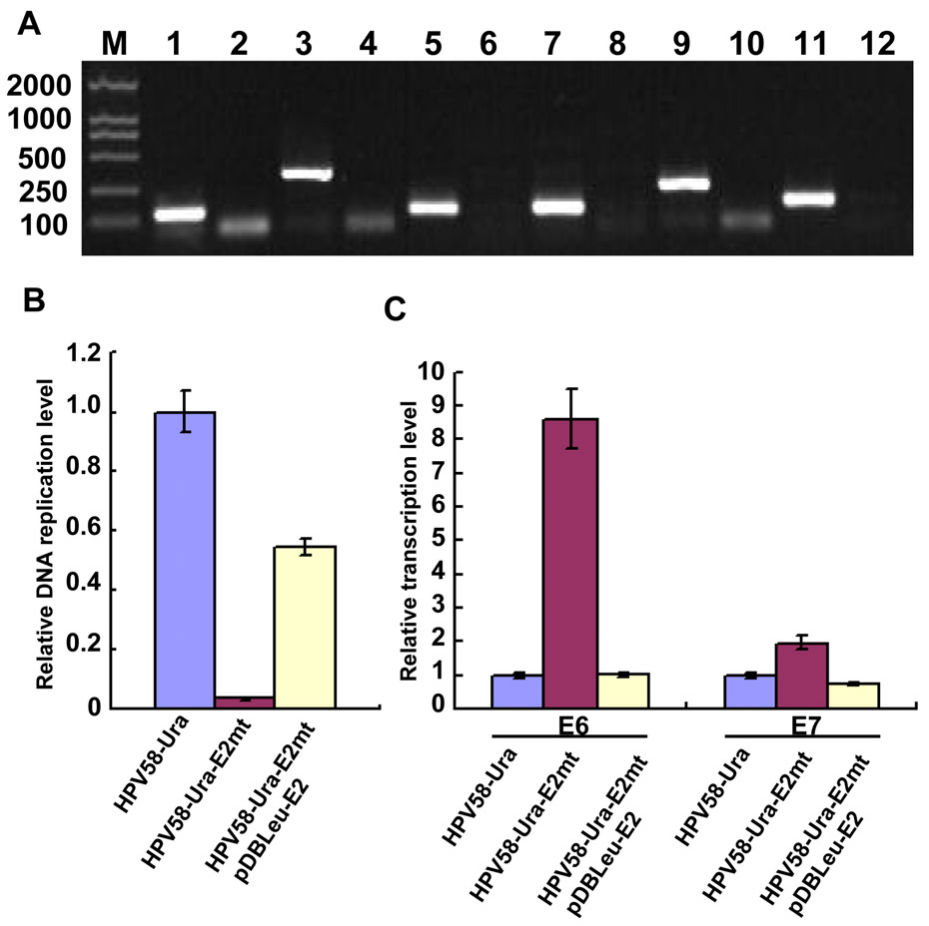
**Transcription of HPV58 genes in *S. cerevisiae***. **A**. RT-PCR analysis of transcriptional expression of HPV58 early and late genes in the HPV58-Ura transformed yeast. E1 (lanes 1, 2), E2 (lanes 3, 4), E6 (lanes 5, 6), E7 (lanes 7, 8), L1 (lanes 9, 10) and L2 (lanes 11, 12) from the cDNA samples. Total RNA was isolated from HPV58-Ura transformed yeast and digested with DNase I. The DNase I treated RNA was amplified to ensure the complete digestion of contaminating viral DNA (lanes 2, 4, 6, 8, 10 and 12). DNase I completely digested RNA were then analysized by RT-PCR (lanes 1, 3, 5, 7, 9 and 11). **B**. Relative replication levels of HPV58 genomes in HPV58-Ura, HPV58-Ura-E2mt and HPV58-Ura-E2mt/pDBLeu-E2 transformed yeast. 18S rDNA was used as internal control. The DNA relative replication levels of HPV58-Ura-E2mt (0.03) and HPV58-Ura-E2mt/pDBLeu-E2 (0.54) are relative to that of HPV58-Ura, which was set to 1.0. Standard deviations are indicated by error bars. **C**. Relative transcription levels of E6 and E7 genes for HPV58-Ura, HPV58-Ura-E2mt and HPV58-Ura-E2mt/pDBLeu-E2. DNase I treated RNA was analysized by qRT-PCR with 18S rRNA as internal control. As for HPV58 genomes have different replication efficiency in the presence or absence of E2 protein (Figure 3B), the transcription levels of E6 and E7 genes were then standardized to the relative DNA replication assay. The E6 and E7 genes transcriptional levels of HPV58-Ura were set to 1.0. The transcription levels of E6 are 8.62 for HPV58-Ura-E2mt and 1.02 for HPV58-Ura-E2mt/pDBLeu-E2. The E7 transcription levels are 1.96 for HPV58-Ura-E2mt and 0.74 for HPV58-Ura-E2mt/pDB Leu-E2 relative to that of HPV58-Ura, which was set to 1.0. Standard deviations are indicated by error bars.

RT-PCR analysis revealed the transcription of both early (E1, E2, E6, E7) and late genes (L1 and L2) of HPV58-Ura in yeast (Figure 3A). To further investigate the transcription levels of viral genes in yeast cells, qRT-PCR was performed separately with gene specific primers. The results revealed that HPV58 early and late genes have different transcription efficiency (Table 2).

**Table 2:** Transcription levels of HPV58 genes in yeast

Gene	mRNA Copies/μg total RNA*
E1	6.54×10^5^ ± 1.03×10^4^
E2	2.42×10^4^ ± 1.83×10^3^
E6	2.48×10^4^ ± 1.77×10^3^
E7	3.75×10^5^ ± 2.68×10^4^
L1	2.08×10^5^ ± 1.73×10^4^
L2	1.34×10^6^ ± 1.24×10^4^

**Note: **Total RNA isolated from yeast cells were digested with DNase I and converted into cDNAs by reverse transcription. The standard curve was generated by serially diluted pEGFPN1-HPV58-Ura. The levels of the viral gene transcripts examined in per microgram total RNA were analyzed by qRT-PCR.

*Values are mean ± standard deviation.

To explore the regulatory function of E2 protein on viral gene transcription, E6 and E7 genes transcription levels in the three yeast transformants were compared by qRT-PCR. We first compared the DNA relative replication levels of HPV58 genome in yeast by qPCR. With the relative replication level of HPV58-Ura set to 1.0, DNA relative replication levels were 0.03 for HPV58-Ura-E2mt and 0.54 for HPV58-Ura-E2mt/pDBLeu-E2 (Figure 3B).

Then qRT-PCR was performed to compare the transcription levels of E6 and E7 genes. Because wild type HPV58 genome and E2 mutant HPV58 genome have different replication levels, the relative transcription levels were divided by the value of relative replication levels. With E6 and E7 transcripion levels of the HPV58-Ura transformants set to 1.0, the relative transcription levels of E6 were 8.62 for HPV58-Ura-E2mt and 1.02 for HPV58-Ura-E2mt/pDBLeu-E2. The E7 relative transcription levels were 1.96 for HPV58-Ura-E2mt and 0.74 for HPV58-Ura-E2mt/pDBLeu-E2 (Figure 3C). Therefore, E2 protein could suppress the E6 and E7 genes transcription levels when HPV58 genome persists episomally in yeast.

## Discussion

Previous reports have shown that HPV1, 6, 11, 16, 18 and 31 can replicate their genomes as episomal plasmids in yeast. The genomes of HPV6, 16 and 31 are mitotically stable in yeast, however HPV1, 11, and 18 are unstable in yeast [7,8,12]. In the present study, we showed that HPV58 genome, consistent with the HPV6, 16 and 31, can replicate stably as an episomal plasmid in yeast.

Stable maintenance through replication of extrachromosomal DNA in yeast requires autonomous replication sequences (ARS) [13,14], and centromeric elements (CEN) [15]. There is a 10 of 11 nucleotide match to the consensus core ARS sequence (CAS) (5'-[A/T]TTTAT[A/G]TTT[A/T]-3') in the HPV58 genome (accession no. D90400) at nucleotides 1225 to 1216. Yeast origin recognition complex (ORC) may bind to the CAS-like element and initiate viral genome replication. It has generally been thought that replication of papillomaviruses is dependent upon the presence of E1, a DNA helicase, and E2, a transcription and maintenance factor [16,17]. E1 and E2 protein, perhaps in conjunction with CAS-like elements, conduct the replication of HPV58 genome in yeast.

Replication of genomes in yeast is a common feature of HPVs. Different HPV types showed different genome stability in yeast cells. Two kinds of mechanisms may control the mitotic stability of the HPV58 genomes in yeast. First, HPV58 sequences may contain *cis*-elements that can substitute for the CEN required for the maintenance of extrochromosomal DNA in *S. cerevisiae*. The second mechanism may be the function of HPV58 E2 protein, a maintenance protein that can tether viral genomes to mitotic chromosomes in dividing cells [18,19]. Angeletti et al. (2002) reported that the HPV16 genome can replicate stably in yeast cells with the E2 gene interrupted by introducing stop codons. Kim et al (2005) also identified the CEN-like *cis*-elements in the HPV16 genome in yeast and mammalian cells and concluded that the maintenance of the HPV16 genome depends on the CEN-like *cis*-elements, perhaps in conjunction with E2 protein. HPV16 can maintain the mitotic stability of its genome in an E2-independent manner in yeast and mammalian cells [7,20,21].

In contrast to HPV16 genome, the maintenance of HPV58 genome depends on E2 protein. The wild-type HPV58 genome can replicate stably in transformed yeast cells. However, with the HPV58 E2 gene interrupted by mutation, HPV58-Ura-E2mt genome was unstable in yeast. Introduction of pDBLeu-E2 and re-expression of E2 protein restored the stability of HPV58-Ura-E2mt genome in yeast cells. Therefore, the maintenance and faithful segregation of HPV58 genome depends on E2 protein. The HPV58 genome has no CEN-like *cis*-elements. This specific maintenance pattern of the HPV58 genome may provide a possible way to interfere with the early stage of HPV58 infection by blocking the expression of E2 protein.

Previous reports of the HPV/yeast system focused on the replication of HPV DNA. In fact, the HPV/yeast system could be a model for the study of HPV gene transcription. Although we failed to detect the mRNA of HPV58 early and late genes by Northern blot, which may be due to the low transcription levels of viral genes in yeast. RT-PCR results revealed the transcription of HPV58 early and late genes in yeast. Moreover, qRT-PCR analysis of transcription regulation function of E2 protein revealed both E6 and E7 mRNA levels were upregulated with HPV58-Ura-E2mt introduced into yeast. However, with re-expression of E2 protein, the mRNA levels were downregulated. These results confirm that E2 protein can suppress E6 and E7 genes transcription when HPV58 genome persists episomally in yeast.

Bechold et al (2003) reported that E2 protein had no affect on E6/E7 expression in mammalial cells [22]. Bouvard et al (1994) reported that HPV16 and 18 E2 protein could activate their early promoter in CAT luciferase reporter plasmid. However, overexpression of E2 repressesd the early promoter[23]. These reports are different from our data that E2 protein repressed early promoter transcription in yeast. The reasons should be the different cell lines used, different expression levels of E2 protein and different conformation of viral minichromosomes in cells, especially the chromatin conformation of long control region (LCR).

The early promoter of HPV locates in the LCR of the viral genome [24]. The LCR has four E2 protein binding sites (E2BS). E2BS4 is far from the TATA box. The other three E2BS (E2BS1, E2BS2 and E2BS3) are proximal to the TATA box. E2 protein binding to E2BS4, which is far from TATA box, stimulates the transcription level of E6 and E7. However, binding of E2 protein to the other three E2BS represses transcription through steric hindrance of the interaction with the transcriptional initiation factor TFIID at the proximal TATA box [25,26]. Binding of E2 protein to a specific E2BS is determined by the genome status. HPV58 E2 protein might interact with the E2BS proximal to the TATA box when the HPV58 genome is maintained as an episomal plasmid in yeast.

## Conclusions

The HPV58/yeast system we used was able to direct the stable replication of the HPV58 genome and induce the transcription of the early and late genes. As compared with maintenance of the HPV16 genome, HPV58 genome strictly depends on E2 protein. E2 protein can supress the transcription of E6 and E7 genes. With its ease of handling and similarity to mammalian system, the yeast system we described is useful for future study of HPV58 genome replication and the regulatory mechanism of viral gene transcription.

## List of abbreviations

ARS: autonomously replicating sequences; CEN: centromeric elements; HPV: Human papillomavirus; LCR: long control region; qPCR: quantitative PCR; qRT-PCR: quantitative RT-PCR; RT-PCR: reverse transcription PCR;

## Competing interests

The authors declare that they have no competing interests.

## Authors' contributions

JL constructed the HPV58-Ura-E2mt, pDBLeu-E2, participated in Sountern blot, Northern blot, Western blot, DNA stability assay and drafted the manuscript. XW constructed HPV58-Ura, participated in Western blot, DNA stability assay and PCR. JL and HW participated in Southern blot and DNA stability assay. XLZ participated in yeast transformation and DNA stability assay. WT and YDS helped to perform the real time quantitative PCR, Southern blot and Northern blot. XW participated in primers and probe design. XPY gave advices on the design of this study. WMZ designed the study and drafted the manuscript. All authors read and approved the final manuscript.
